# Supplementary material for: Linear infrastructure drives biotic homogenization among bird species of a tropical dry forest
Source: PLoS One. 2025 Apr 16;20(4):e0302756. doi: 10.1371/journal.pone.0302756 (PMC12002429; doi:10.1371/journal.pone.0302756)
Supplement: Supplementary Table 1 — (DOCX) [file pone.0302756.s001.docx]

Title: Linear infrastructure drives biotic homogenization among bird species of a tropical dry forest

Authors: Dishane K Hewavithana, Devaka K Weerakoon, Mayuri R Wijesinghe, Christopher A Searcy

Supplementary Table 1: Checklist of bird species recorded across the 80 survey points (BrR- Breeding resident; Pro: Endemic- Proposed endemic; WV- Winter visitor; VU- Vulnerable; NT- Near Threatened; LC- Least Concern; NE- Not Evaluated).

| **Family** | **Scientific Name** | **English Name** | **SpS** | **CoS** |
| --- | --- | --- | --- | --- |
| Accipitridae | *Accipiter badius* | Shikra | BrR | LC |
|  | *Accipiter virgatus* | Besra | BrR | VU |
|  | *Haliaeetus leucogaster* | White-bellied Sea-eagle | BrR | LC |
|  | *Haliastur indus* | Brahminy Kite | BrR | LC |
|  | *Ichthyophaga ichthyaetus* | Grey-headed Fish-eagle | BrR | NT |
|  | *Spilornis cheela* | Crested Serpent Eagle | BrR | LC |
|  | *Spizaetus cirrhatus* | Changeable Hawk Eagle | BrR | LC |
| Aegithinidae | *Aegithina tiphia* | Common Iora | BrR | LC |
| Alaudidae | *Mirafra affinis* | Rufous-winged Bushlark | BrR | LC |
| Alcedinidae | *Alcedo atthis* | Common Kingfisher | BrR | LC |
|  | *Ceyx erithacus* | Oriental Dwarf Kingfisher | BrR | NT |
|  | *Halcyon smyrnensis* | White-throated Kingfisher | BrR | LC |
|  | *Pelargopsis capensis* | Stork-billed Kingfisher | BrR | LC |
| Apodidae | *Cypsiurus balasiensis* | Asian Palm Swift | BrR | LC |
| Bucerotidae | *Anthracoceros coronatus* | Malabar Pied Hornbill | BrR | LC |
|  | *Ocyceros gingalensis* | Sri Lanka Grey Hornbill | **Endemic** | LC |
| Campephagidae | *Coracina macei* | Large Cuckooshrike | BrR | LC |
|  | *Coracina melanoptera* | Black-headed Cuckooshrike | BrR | LC |
|  | *Pericrocotus cinnamomeus* | Small Minivet | BrR | LC |
|  | *Tephrodornis pondicerianus* | Common Woodshrike | **Endemic** | LC |
| Caprimulgidae | *Caprimulgus asiaticus* | Common Nightjar | BrR | LC |
|  | *Caprimulgus atripennis* | Jerdon’s Nightjar | BrR | LC |
| Charadriidae | *Vanellus indicus* | Red-wattled Lapwing | BrR | LC |
| Chloropseidae | *Chloropsis aurifrons* | Golden-fronted Leafbird | BrR | LC |
|  | *Chloropsis jerdoni* | Blue-winged Leafbird | BrR | LC |
| Cisticolidae | *Prinia hodgsonii* | Grey-breasted Prinia | BrR | LC |
|  | *Prinia inornata* | Plain Prinia | BrR | LC |
|  | *Prinia socialis* | Ashy Prinia | BrR | LC |
|  | *Prinia sylvatica* | Jungle Prinia | BrR | LC |
| Columbidae | *Chalcophaps indica* | Emerald Dove | BrR | LC |
|  | *Ducula aenea* | Green Imperial Pigeon | BrR | LC |
|  | *Streptopelia chinensis* | Spotted Dove | BrR | LC |
|  | *Treron bicincta* | Orange-breasted Green-pigeon | BrR | LC |
|  | *Treron pompadora* | Pompadour Green-pigeon | **Endemic** | LC |
| Coraciidae | *Coracias benghalensis* | Indian Roller | BrR | LC |
| Corvidae | *Corvus levaillantii* | Large-billed Crow | BrR | LC |
| Cuculidae | *Cacomantis sonneratii* | Banded Bay Cuckoo | BrR | NT |
|  | *Centropus sinensis* | Greater Coucal | BrR | LC |
|  | *Cuculus micropterus* | Indian Cuckoo | SU | NE |
|  | *Eudynamys scolopacea* | Asian Koel | BrR | LC |
|  | *Surniculus lugubris* | Drongo Cuckoo | BrR | NT |
| Dicaeidae | *Dicaeum agile* | Thick-billed Flowerpecker | BrR | NT |
|  | *Dicaeum erythrorhynchos* | Pale-billed Flowerpecker | BrR | LC |
| Dicruidae | *Dicrurus caerulescens* | White-bellied Drongo | BrR | LC |
|  | *Dicrurus paradiseus* | Great Racket-tailed Drongo | BrR | NT |
| Estrididae | *Lonchura punctulata* | Scaly-breasted Munia | BrR | LC |
|  | *Lonchura striata* | White-rumped Munia | BrR | LC |
| Hemiprocnidae | *Hemiprocne coronata* | Crested Treeswift | BrR | LC |
| Laniidae | *Lanius cristatus* | Brown Shrike | WV | NE |
| Meropidae | *Merops leschenaulti* | Chestnut-headed Bee-eater | BrR | LC |
|  | *Merops orientalis* | Green Bee-eater | BrR | LC |
|  | *Merops philippinus* | Blue-tailed Bee-eater | WV | NE |
| Monarchidae | *Hypothymis azurea* | Black-naped Monarch | BrR | LC |
|  | *Terpsiphone paradisi* | Asian Paradise- flycathcher | BrR/WV | LC |
| Motacillidae | *Dendronanthus indicus* | Forest Wagtail | WV | NE |
| Muscicapidae | *Copsychus malabaricus* | White-rumped Shama | BrR | LC |
|  | *Copsychus saularis* | Oriental Magpie Robin | BrR | LC |
|  | *Cyornis tickelliae* | Tickell’s Blue Flycatcher | BrR | LC |
|  | *Luscinia brunnea* | Indian Blue Robin | WV | NE |
|  | *Muscicapa daurica* | Asian Brown Flycatcher | WV | NE |
| Nectariniidae | *Nectarina asiatica* | Purple Sunbird | BrR | LC |
|  | *Nectarina lotenia* | Loten’s Sunbird | BrR | LC |
|  | *Nectarina zeylonica* | Purple-rumped Sunbird | BrR | LC |
| Oriolidae | *Oriolus xanthornus* | Black-hooded Oriole | BrR | LC |
| Phasianidae | *Gallus lafayetii* | Sri Lanka Junglefowl | **Endemic** | LC |
|  | *Pavo cristatus* | Indian Peafowl | BrR | LC |
| Picidae | *Celeus brachyurus* | Rufous Woodpecker | BrR | LC |
|  | *Chrysocolaptes stricklandi* | Layard’s Flameback | **Endemic** | LC |
|  | *Dendrocopus nanus* | Brown-capped Pygmy Woodpecker | BrR | LC |
|  | *Dinopium benghalense* | Black-rumped Flameback | BrR | LC |
| Pittidae | *Pitta brachyura* | Indian Pitta | WV | NE |
| Podargidae | *Batrachostomus moniliger* | Frogmouth | BrR | LC |
| Psittacidae | *Psittacula eupatria* | Alexandrine Parakeet | BrR | LC |
|  | *Psittacula krameri* | Rose-ringed Parakeet | BrR | LC |
| Pycnonotidae | *Pycnonotus cafer* | Red-vented Bulbul | BrR | LC |
|  | *Pycnonotus luteolus* | White-browed Bulbul | BrR | LC |
|  | *Pycnonotus melanicterus* | Black-capped Bulbul | **Endemic** | LC |
| Ramphastidae | *Megalaima haemacephala* | Coppersmith Barbet | BrR | LC |
|  | *Megalaima rubricapilla* | Crimson-fronted Barbet | **Endemic** | LC |
|  | *Megalaima zeylanica* | Brown-headed Barbet | BrR | LC |
| Rhipiduridae | *Rhipidura aureola* | White-browed Fantail | BrR | LC |
| Sittidae | *Sitta frontalis* | Velvet-fronted Nuthatch | BrR | LC |
| Strigidae | *Ketupa zeylonensis* | Brown Fish Owl | BrR | LC |
|  | *Ninox scutulata* | Brown Hawk Owl | BrR | LC |
|  | *Strix leptogrammica* | Brown Wood Owl | BrR | NT |
| Sturnidae | *Acridotheres tristis* | Common Myna | BrB | LC |
| Sylviidae | *Acrocephalus dumetorum* | Blyth’s Reed Warbler | WV | NE |
|  | *Orthotomus sutorius* | Common Tailorbird | BrR | LC |
|  | *Phylloscopus magnirostris* | Large-billed Leaf Warbler | WV | NE |
|  | *Phylloscopus trochiloides* | Greenish Warbler | WV | NE |
| Timalidae | *Pellorneum fuscocapillum* | Sri Lanka Brown-capped Babbler | **Endemic** | LC |
|  | *Rhopocichla atriceps* | Dark-fronted Babbler | BrR | LC |
|  | *Turdoides affinis* | Yellow-billed Babbler | BrR | LC |
| Turdidae | *Zoothera citrina* | Orange-headed Thrush | WV | NE |
| Zosteropidae | *Zosterops palpebrosus* | Oriental White-eye | BrR | LC |
